# Editorial: Harvesting Plant and Microbial Biodiversity for Sustainably Enhanced Food Security

**DOI:** 10.3389/fpls.2018.00042

**Published:** 2018-01-31

**Authors:** Laurent Laplaze, Francesca Sparvoli, Khaled Masmoudi, Charles T. Hash

**Affiliations:** ^1^Laboratoire Mixte International Adaptation des Plantes et Microorganismes Associés Aux Stress Environnementaux, Laboratoire Commun de Microbiologie IRD/ISRA/UCAD, Dakar, Senegal; ^2^UMR DIADE, Institut de Recherche pour le Développement, Université de Montpellier, Montpellier, France; ^3^Istituto di Biologia e Biotecnologia Agraria, Dipartimento di Scienze Bio-Agroalimentari, Consiglio Nazionale delle Ricerche, Milan, Italy; ^4^Department of Aridland, United Arab Emirates University, Al-Ain, United Arab Emirates; ^5^International Crop Research Institute for the Semi-Arid Tropics, Niamey, Niger

**Keywords:** food security, breeding, biodiversity, inoculation, microbiome, drought tolerance, Salinization, climate change

According to the United Nations, the World population will reach 9 billion by 2050, with the majority of this growth occurring in developing countries. More than half of global population growth is expected to occur in Africa. On the other hand, one in nine of the World's population suffers from chronic hunger, the vast majority of which live in developing countries (FAO et al., 2015). We therefore need to find new and sustainable solutions to feed this increasing population and alleviate the predicted negative impact of global changes on crop production. This e-Book summarize current research to improve food security and livelihoods in rural communities, reduce vulnerability, increase resilience, and mitigate land degradation in developing countries.

Several reviews and articles addressed the current status and strategies to deal with the major abiotic factors limiting crop production. Sultan and Gaetani review the current predictions of future climate in West Africa, one of the most dynamic area for demographic growth, as well as its expected impact on crop production and scenario for adaptation. Drought episodes are expected to occur more frequently and Ndour et al. review how structural-functional plant models can be used to understand water acquisition and to breed varieties with increase tolerance to water deficit. Beside, about 6% of the arable land is affected by salinization worldwide. The problem is mostly concentrated in arid and semiarid regions, where it seriously threatens agricultural sustainability and food security. Salinity induces a rapid osmotic stress that reduces shoot growth, and a slower ionic stress that accelerates senescence of older leaves (Munns and Tester, 2008). Adaptation to salinity is a quantitative character, which is controlled by different genetic pathways (DeRose-Wilson and Gaut, 2011). Hanin et al. discuss how our current knowledge on sodium accumulation and transport and how beneficial plant-microbe interactions can be used to create varieties and agricultural practices to improve yield in salt-affected areas. Phosphorus availability is another major limiting factor for crop production and the limited amount of available high quality rock phosphate deposits to make fertilizer reinforce the need to find alternative strategies to improve P acquisition and use efficiency. Gemenet et al. discuss the various strategies that could be used to achieve these goals for sorghum and pearl millet in the context of West Africa. Vandamme et al. show that genetic diversity exists in rice for P accumulation in seeds and could be used to limit the removal of P from agricultural soil. Indeed, keeping P in the biomass that can be recycled back to the soil rather than the grain could limit P depletion.

Clearly, there is a need to characterize and exploit available plant biodiversity to increase production and sustainability in agrosystems. In their review, Mondal et al. discuss new breeding strategies that could be used to exploit the genetic diversity in wheat. Pearl millet is an important crop for food security in arid and semi-arid areas. The characterization of the genetic diversity of cultivated pearl millet in Senegal by Diack et al. reveals a genetic differentiation between early- and late-flowering varieties and a very large and untapped potential for breeding.

Root traits (including root-beneficial microbe interactions) are potential new targets to breed new varieties with improved resource use efficiency. The review by Schmidt et al. discusses how domestication and modern breeding indirectly affected root development and rhizosphere ecology in maize and how these could be new targets to create varieties with more efficient resource acquisition. Similarly, Passot et al. analyzed early root development and root anatomy in pearl millet. They demonstrate that there is diversity for root traits such as root growth or branching that could be used for breeding.

Beneficial microbes are another potential lever to improve production and reduce the use of fertilizers and pesticides. While many studies have revealed the potential beneficial impact of microbes, little are used in agriculture. The review article by Parnell et al. presents the factors that need to be taken into account to develop inoculants for agriculture and give some examples of products available and their use. Two original research papers report the impact of microorganisms that could be used as bio-fertilizer. Zhang et al. report the impact of *Trichoderma longibrachiatum* T6 on wheat tolerance to salt stress, and provide evidence that the beneficial effect involves the antioxidative defense system of the plant. Similarly, Akram et al. provide evidences that *Staphylococcus sciuri* strain SAT-17 alleviates salt-induced cellular damages in maize plants and enhances growth. Pitzschke et al. also report that seeds of the hardy crop quinoa contain bacteria of the genus *Bacillus* that could change the host's redox status and induce a primed state that might enhance plant tolerance to abiotic stresses.

Ecological intensification of agroecosystems could also be achieved using plant association. For instance, Wahbi et al. show that intercropping wheat and faba bean gave better crop productivity than rotation practice, impacting soil microbial functionalities. Tropical trees of the *Casuarinaceae* family can enter nitrogen-fixing symbiosis with the soil actinomycete *Frankia* sp. and are widely used to rehabilitate including salinized soils. Ngom et al. show that salt stress reduces symbiosis formation in *Casuarina glauca* and *C. equisetifolia* and that *Frankia* nitrogen-fixation efficiency rather than *in vitro* salt tolerance is important to improve salt tolerance of inoculated plants.

Finally, two original research articles deal with crop quality characters for food and industrial uses. Neglected and underutilized crops might represent an alternative to current staple crops, especially in marginal lands such as those of the arid and semi-arid regions of sub-Saharan Africa (Naylor et al., 2004). Ghebrehiwot et al evaluated the use of flours from *Eragrostis curvula*, a wild relative of tef (*Eragrostis tef*), for preparing injera, a typical sour bread consumed as staple food in Ethiopia and Eritrea. Cassava is an important crop for food security and a major source of starch for industry (Li et al., 2017). In their article, Karlström et al. report that cassava varieties with amylose-free starch have no limited impact on yield and could therefore be planted to increase the revenues of farmers.

## Author contributions

All authors listed have made a substantial, direct, and intellectual contribution to the work, and approved it for publication.

### Conflict of interest statement

The authors declare that the research was conducted in the absence of any commercial or financial relationships that could be construed as a potential conflict of interest.
